# A conserved Eph family receptor-binding motif on the gH/gL complex of Kaposi’s sarcoma-associated herpesvirus and rhesus monkey rhadinovirus

**DOI:** 10.1371/journal.ppat.1006912

**Published:** 2018-02-12

**Authors:** Anna K. Großkopf, Armin Ensser, Frank Neipel, Doris Jungnickl, Sarah Schlagowski, Ronald C. Desrosiers, Alexander S. Hahn

**Affiliations:** 1 German Primate Center - Leibniz Institute for Primate Research, Göttingen, Germany; 2 Universitätsklinikum Erlangen, Institute for Clinical and Molecular Virology, Erlangen, Germany; 3 Miller School of Medicine, University of Miami, Miami, United States of America; Louisiana State University Health Sciences Center, UNITED STATES

## Abstract

Kaposi’s sarcoma-associated herpesvirus (KSHV) is a human oncogenic virus associated with Kaposi’s sarcoma and two B-cell malignancies. The rhesus monkey rhadinovirus (RRV) is a virus of nonhuman primates that is closely related to KSHV. Eph family receptor tyrosine kinases (Ephs) are cellular receptors for the gH/gL glycoprotein complexes of both KSHV and RRV. Through sequence analysis and mutational screens, we identified conserved residues in the N-terminal domain of KSHV and RRV glycoprotein H that are critical for Eph-binding *in vitro*. Homology-based structural predictions of the KSHV and RRV gH/gL complexes based on the Epstein-Barr-Virus gH/gL crystal structure located these amino acids in a beta-hairpin on gH, which is likely stabilized by gL and is optimally positioned for protein-protein interactions. Guided by these predictions, we generated recombinant RRV and KSHV strains mutated in the conserved motif as well as an RRV gL null mutant. Inhibition experiments using these mutants confirmed that disruption of the identified Eph-interaction motif or of gL expression resulted in complete detargeting from Ephs. However, all mutants were infectious on all cell types tested, exhibiting normal attachment but a reduction in infectivity of up to one log order of magnitude. While Eph-binding-negative RRV mutants were replication-competent on fibroblasts, their infectivity was comparatively more reduced on endothelial cells with a substantial subpopulation of endothelial cells remaining resistant to infection. Together, this provides evidence for a cell type-specific use of Ephs by RRV. Furthermore, our results demonstrate that gL is dispensable for infection by RRV. Its deletion caused a reduction in infectivity similar to that observed after mutation of Eph-binding residues in gH. Our findings would be compatible with an ability of KSHV and RRV to use other, less efficient entry mediators in lieu of Ephs, although these host factors may not be uniformly expressed by all cells.

## Author summary

In immunocompromised individuals in general and in the context of HIV infection in particular, KSHV is a major cause of cancer and B-cell proliferative malignancies. We identified and mutated conserved residues in the N-terminal domain of the gH/gL glycoprotein complex of KSHV and the related monkey virus RRV that are critical for the interaction with cellular receptors from the Eph family. These findings provide important insight into the function of the γ-herpesviral entry machinery. Using recombinant KSHV and RRV carrying these mutations, we demonstrated that while not strictly essential, gH/gL-Eph interactions are important for efficient infection—for RRV also in a cell-specific manner—but not for attachment of KSHV and RRV. The Eph-detargeted virus mutants described in this study can be used to further dissect the requirements for KSHV and RRV entry and to identify potential alternative entry mediators. Domains and residues on the viral glycoproteins with critical roles in receptor recognition, such as the Eph-binding motif described in this paper, can be informative for the design of inhibitory monoclonal antibodies.

## Introduction

Kaposi’s sarcoma-associated herpesvirus (KSHV), the etiological agent of Kaposi’s Sarcoma [1], is also closely associated with two B-cell malignancies, namely the primary effusion lymphoma [2] and the plasmablastic variant of multicentric Castleman’s disease [3](reviewed in [4]). Together with the rhesus monkey rhadinovirus (RRV), a closely related herpesvirus of rhesus macaques, KSHV belongs to the rhadinovirus, or γ2, genus of herpesvirus [5]. Two RRV isolates, RRV isolate 26–95 [6] and RRV isolate 17577 [7], representing different subtypes have been characterized. RRV shares many biological features with KSHV and is therefore regarded as an *in vivo* model system for many aspects of γ2-herpesvirus biology [8–10] (reviewed in [11]). With regard to entry into target cells, some differences but also strong similarities exist between KSHV and RRV. A prominent difference is the interaction with integrins, which is shared by most herpesviruses (reviewed in [12]). In the case of KSHV, interaction with integrins is mediated through glycoprotein B (gB) [13], the conserved herpesviral fusion executor. Detectable interaction of RRV with integrins has not been observed, at least not via the same glycoprotein or mechanism [14]. On the other hand, the interaction of the respective gH/gL glycoprotein complex of KSHV and RRV with members of the Ephrin receptor tyrosine kinase (RTK) family of proteins (Ephs) is a conserved feature in the entry process of both rhadinoviruses. Whether the interaction of rhadinoviral gH/gL with Ephs also promotes fusion is so far unclear. In contrast, contribution to virus endocytosis, trafficking, and establishment of infection has been described by several reports [15–19]. KSHV binds EphA2 with high affinity and only exhibits very weak interactions with other A-type Ephs [15,16]. Despite very divergent primary sequences of gH and gL of RRV 26–95 and 17577, both isolates were found to interact with a broad spectrum of A- and B-type Eph receptors and to bind EphB3 with the highest avidity [16]. In addition, both KSHV and RRV require the presence of gH as well as gL in the gH/gL complex for Eph-interaction.

While we recently have shown that the binding site for the KSHV gH/gL complex on EphA2 is similar to that of natural ephrin ligands [20], the corresponding interaction site on the gH/gL complex has remained elusive until now. Recent structure-function analyses of other herpesviruses suggested different domains of the herpesviral gH as determinants for entry into target cells. For instance, the N-terminal tip of domain (D)I of the varicella-zoster virus (VZV) homolog was shown to play a role in virus entry and fusion, as well as VZV skin tropism [21]. Similarly, in Epstein-Barr-Virus (EBV) infection, DI of gH was described as a determinant of membrane fusion activity and gB interaction [22]. Additionally, the monoclonal anti-gH/gL antibody E1D1 which inhibits EBV membrane fusion with epithelial cells was shown to bind to the tip of the gH/gL DI through interaction with gL residues [23]. Other studies suggested a role of residues in DII of EBV gH in gB-mediated membrane fusion, which is mediated by an integrin-binding ‘KGD’ motif located in the central region of the gH/gL complex [24]. Therefore, the site of the Eph interaction cannot easily be inferred from similar receptor interactions by other viruses. A detailed analysis of the evolutionarily conserved interaction with Eph family receptors and the regions on the gH/gL complex involved in this interaction would further our general understanding of the herpesviral gH/gL glycoprotein complex.

The actual contribution of the Eph receptor interaction to infection of different cell types by KSHV and RRV also deserves further analysis. Inhibition of KSHV infection by blocking of the Eph interaction ranged from almost complete to around twofold in previous studies depending on experimental setup and cell type [16,18]. These findings raised the question whether the interaction with Eph family receptors by KSHV and RRV is obligatory, obligatory only on certain cell types, or simply has a very strong enhancing effect on infection. Such a strong enhancing effect, depending on the setting, may still make this interaction obligatory to achieve detectable infection. Studying entry of KSHV and RRV is complicated by the fact that the Eph family comprises 14 homologous members in both humans and rhesus monkeys, by the complexity of the Eph-ephrin signaling network, and by the physiological importance of this RTK family. Various members of the Eph family were shown to play prominent roles in a wide range of physiological and pathological events, including the regulation of developmental processes, angiogenesis, cancer, and inflammation (reviewed in [25–27]). Blocking or ablating expression of Ephs may have strong effects on some cell types, exemplified by the dependence of several tumor cell lines on EphA2 expression for ongoing growth *in vitro* [28,29]. Furthermore, RRV binds very promiscuously to many of the 14 A- and B-type Ephs, impeding clean knockout experiments of distinct Ephs by compensatory effects of other members of the family. Even for KSHV, which binds EphA2 very selectively with high affinity, weak interactions with other A-type Ephs were detectable [16] and may confound results. Targeting the viral side of the gH/gL-Eph interaction would overcome this limitation.

Therefore, the aim of this study was first to address the question of whether the conserved interaction with Ephs is based on an equally conserved viral binding motif on gH/gL and to map this interaction site on the gH/gL complex of KSHV and RRV. Second, we sought to use this information to generate mutant KSHV and RRV strains that are unable to interact with Eph family receptors and to evaluate the relative importance of this interaction for infection of different cell types.

## Results

### An evolutionarily conserved motif in domain I of gH is critical for Eph receptor interaction

**Fig 1:**
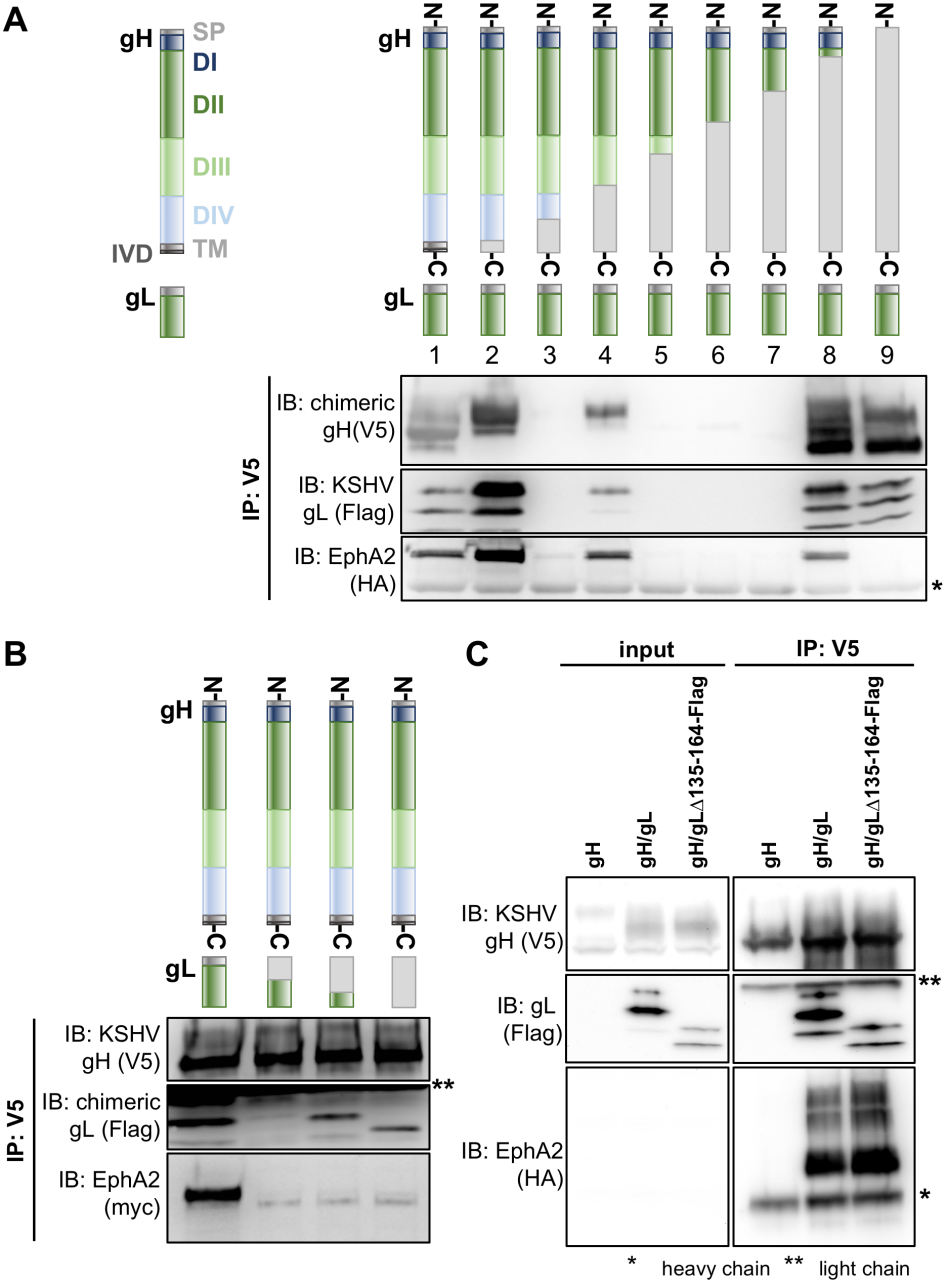
The Eph RTK interaction maps to the N-terminal region of KSHV gH. **A** Co-Immunoprecipitation of depicted V5-tagged RRV/KSHV gH chimeras in complex with KSHV gL-Flag identifies KSHV gH domains necessary for binding of EphA2. Monoclonal antibody to the V5-tag was used for precipitation of complexes. Equal amounts of HA epitope-tagged EphA2 ectodomain was added to each reaction. EphA2, gH, gL were detected using their respective tag. RRV-derived parts of the chimeric constructs are displayed in light grey; lane 1: KSHV gH, lane 9: RRV gH. Four gH chimeras (lanes 3 and 5–7) were not expressed to detectable levels. All chimeras harboring domain I of KSHV gH (lanes 2, 4, 8) were still able to interact with EphA2. **B** Co-Immunoprecipitation of V5-tagged KSHV gH in complex with Flag-tagged RRV/KSHV gL chimeras identifies KSHV gL domains necessary for binding of EphA2. gH-V5/gL-Flag complexes were immunoprecipitated in the presence of full-length EphA2-myc using monoclonal antibody to the V5-tag and precipitates were analyzed by Western blot as in A. RRV-derived parts of the chimeric constructs are displayed in light grey; first lane: KSHV gL, fourth lane: RRV gL. **C** Co-Immunoprecipitation of V5-tagged KSHV gH in complex with a Flag-tagged C-terminally truncated KSHV gL mutant (gLΔ135–164). gH-V5/gL-Flag complexes were immunoprecipitated in the presence of EphA2-HA (ectodomain) using monoclonal antibody to the V5-tag and precipitates were analyzed by Western blot as in A. Full-length KSHV gH/gL serves as a positive control, KSHV gH alone serves as a negative control. Asterisks indicate non-specific bands. Abbreviations: D: domain, TM: transmembrane domain, SP: signal peptide, IVD: intravirion domain, IP: immunoprecipitation, IB: immunoblotting.

**Fig 2:**
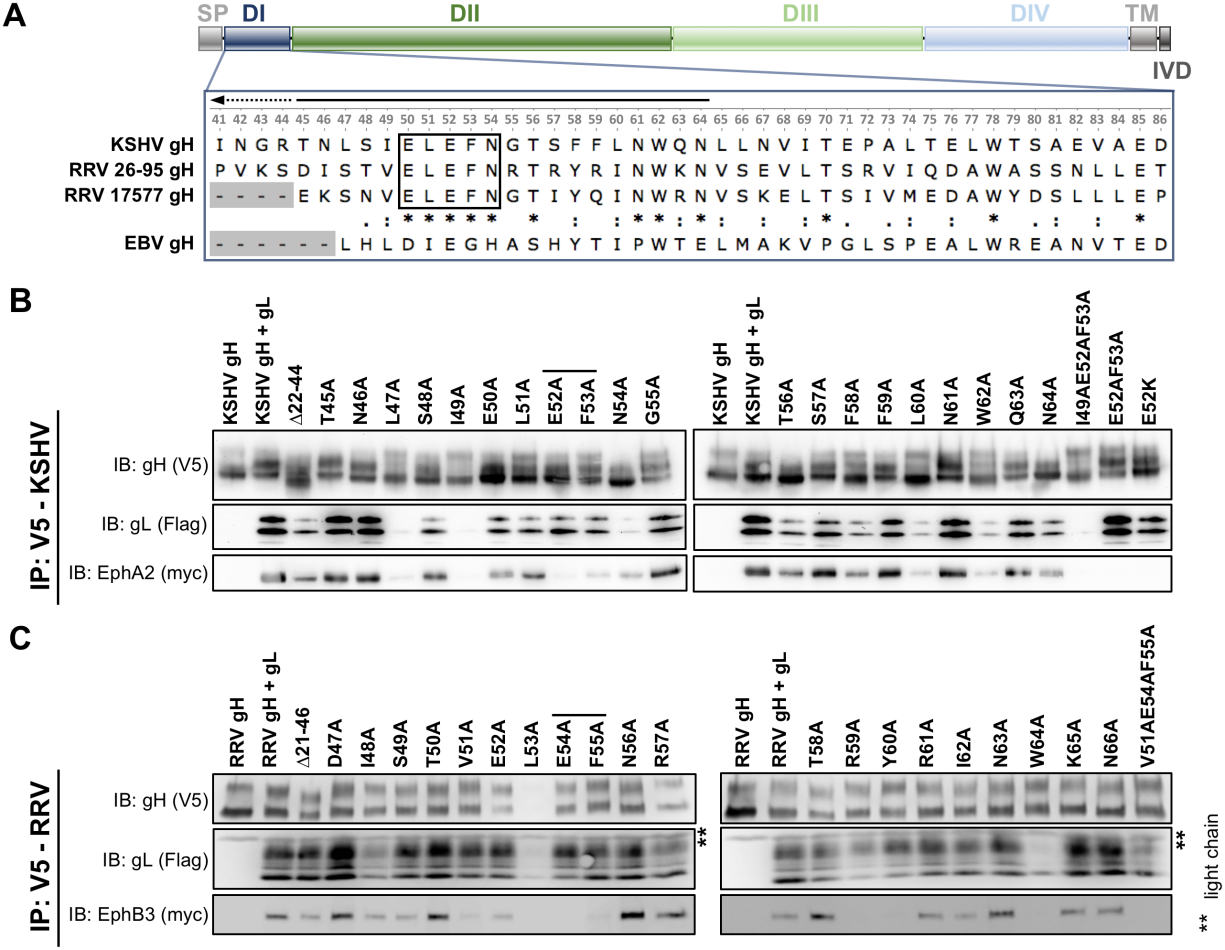
Two amino acids of a conserved E-L-E-F-N motif in the N-terminal region of KSHV and RRV gH are essential for Eph interaction. **A** Domain structure of KSHV and RRV gH. Multiple sequence alignment of domain I of gH of KSHV and the two RRV isolates 26–95 and 17577 (enlarged inset, numbers corresponding to KSHV gH). The EBV gH sequence is included as a reference. **B** Mutational scan of the N-terminal region of KSHV gH identifies EphA2-interacting residues. V5-tagged KSHV gH mutants were co-expressed with Flag-tagged KSHV gL. gH-V5/gL-Flag complexes were immunoprecipitated in the presence of full-length EphA2-myc using monoclonal antibody to the V5-tag and precipitates were analyzed by Western blot. KSHV gH alone serves as negative control. **C** Mutational scan of the N-terminal region of RRV gH identifies EphB3-interacting residues. V5-tagged gH mutants were co-expressed with Flag-tagged RRV gL. gH-V5/gL-Flag complexes were immunoprecipitated in the presence of full-length EphB3-myc using monoclonal antibody to the V5-tag and precipitates were analyzed by Western blot. RRV gH alone serves as negative control. Residues in the conserved E-L-E-F-N motif that are critical for Eph interaction are indicated by black lines. Asterisks indicate non-specific bands. Abbreviations: D: domain, TM: transmembrane domain, SP: signal peptide, IVD: intravirion domain, IP: immunoprecipitation, IB: immunoblotting.

Based on the known structures of the herpes simplex virus type 2 and EBV gH/gL complex [22,30] and the conserved nature of this glycoprotein complex, it can be assumed that gH of KSHV comprises domain I (D I) to domain IV (D IV), a transmembrane domain (TM) and a short C-terminal intravirion domain (IVD). To identify the region of gH/gL critical for Eph receptor interaction, we constructed chimeras composed of different regions derived from either the KSHV or RRV gH primary sequence. Transfected chimeric gH constructs were co-expressed with KSHV gL to form stable gH/gL complexes. Using co-immunoprecipitation assays, we tested the complexes of KSHV/RRV chimeric gH and KSHV gL for their ability to bind EphA2, which is the high affinity Eph family receptor for KSHV gH/gL and exhibits only marginal or no interaction with RRV gH/gL (Fig 1A). Out of the seven tested constructs, three were detected in Western blot analysis (Fig 1A, lane 2, 4, 8). Due to the relatively high sequence diversity of rhadinovirus gH proteins, molecular mass and glycosylation vary slightly between KSHV and RRV derived regions leading to visible shifts in apparent migration of gH chimeras when compared to KSHV gH. All of the chimeras that were expressed, even if only comprising the N-terminal and so-called shoulder region of KSHV gH (Fig 1A, lane 8), were found to complex with KSHV gL and to bind EphA2. In contrast, while full-length RRV gH did form a complex with KSHV gL, this complex did not interact with EphA2 (Fig 1A, lane 9). Likewise, N-terminal KSHV/RRV chimeras of gL or full-length RRV gL did not support binding to EphA2 when co-expressed with full-length KSHV gH in the gH/gL complex (Fig 1B). Additionally, KSHV gH/gLΔ135–164, consisting of full-length KSHV gH and a C-terminal gL truncation mutant, precipitated EphA2 to wild-type levels (Fig 1C). This indicates that the N-terminal domains of KSHV gH and of KSHV gL in the gH/gL complex are essential for the interaction with EphA2. The natural ligands of Ephs, the eight ephrins, interact with their respective receptors through a structurally conserved G-H loop that also exhibits substantial conservation on the amino acid level [31]. We therefore aimed to identify an equally conserved Eph interaction motif on the rhadinoviral gH. First, we performed comparative sequence analysis of the gH proteins of KSHV and the two RRV isolates 17577 and 26–95, comparing the three rhadinoviral gH sequences to that of EBV gH (Fig 2A). Interestingly, we found a highly conserved motif present in all three gH sequences listed here as well as in all RRV and KSHV sequences currently listed in the NCBI database that consists of the five amino acids Glu(E)-Leu(L)-Glu(E)-Phe(F)-Asn(N) (Fig 2A, black rectangle) and is not perfectly conserved in EBV. To investigate the relevance of this E-L-E-F-N motif for the interaction with Eph receptors, a mutational scan of the N-terminal regions of KSHV and RRV gH was performed by substituting single amino acids with alanine. First, we tested the influence of these amino acid substitutions on the stability of the gH/gL heterodimer by immunoprecipitation assays (S1A and S1B Fig). The mutant KSHV and RRV gH/gL complexes were immunoprecipitated via the V5-tagged gH in the absence of recombinant Eph receptors and complexation with gL was assayed by Western blot after normalization for differences in expression levels. Mutations L47A and I49A (KSHV) and W64A (RRV) resulted in a strongly decreased interaction with gL. RRV gH E52A and L53A exhibited a reduced expression and slightly aberrant glycosylation pattern, and L53A also incorporated fully glycosylated gL less efficiently (S1B Fig). The ability of mutant gH/gL complexes to interact with either myc-tagged full-length EphA2 for KSHV gH/gL (Fig 2B), or myc-tagged full-length EphB3 for RRV gH/gL (Fig 2C) was analogously analyzed by immunoprecipitation via the V5-tagged gH and Western blot. This approach identified several point mutations that resulted in a loss of EphA2 or EphB3 interaction, respectively. Among those, the described mutations that lead to a reduced gL interaction represent one group. As loss of gL in the gH/gL complex in itself is sufficient to abolish Eph interaction (Fig 2B and 2C, first lane), the reduced interaction of these gH point mutants with Eph receptors can most likely be attributed to a loss of the gL interaction. We therefore aimed to identify gH point mutants that exhibited a normal expression level and glycosylation pattern and incorporated gL to wt levels. Notably, we identified two amino acids in the conserved E-L-E-F-N motif (Glu52 and Phe53 for KSHV, Glu54 and Phe55 for RRV) whose side chains are essential for Eph receptor interaction but at the same time dispensable for gL binding (Fig 2B and 2C, indicated by black lines). Single point mutations of other amino acids adjacent to the described E-L-E-F-N motif, specifically V51A, R59A and Y60A (RRV) as well as L60A and W62A (KSHV) also abrogated binding of Eph receptors while apparently causing only a minor decrease in gL association. This might indicate additional, direct interaction with EphB3 or EphA2, respectively, at those positions.

Combination of mutations E52A and F53A of KSHV gH completely abrogated EphA2 binding without affecting gL association or expression (Fig 2B, second lane from the right, S1C Fig). Likewise, a V51A-E54A-F55A triple mutant of RRV gH was negative for EphB3 interaction, while maintaining the capacity to bind RRV gL and a normal expression level (Fig 2C, rightmost lane, S1C Fig). Finally, introducing a charge reversal by mutating glutamic acid at position 52 of KSHV gH to lysine (E52K) also abrogated EphA2 binding (Fig 2B, rightmost lane), implicating a critical role of this negative charge in the interaction.

**Fig 3:**
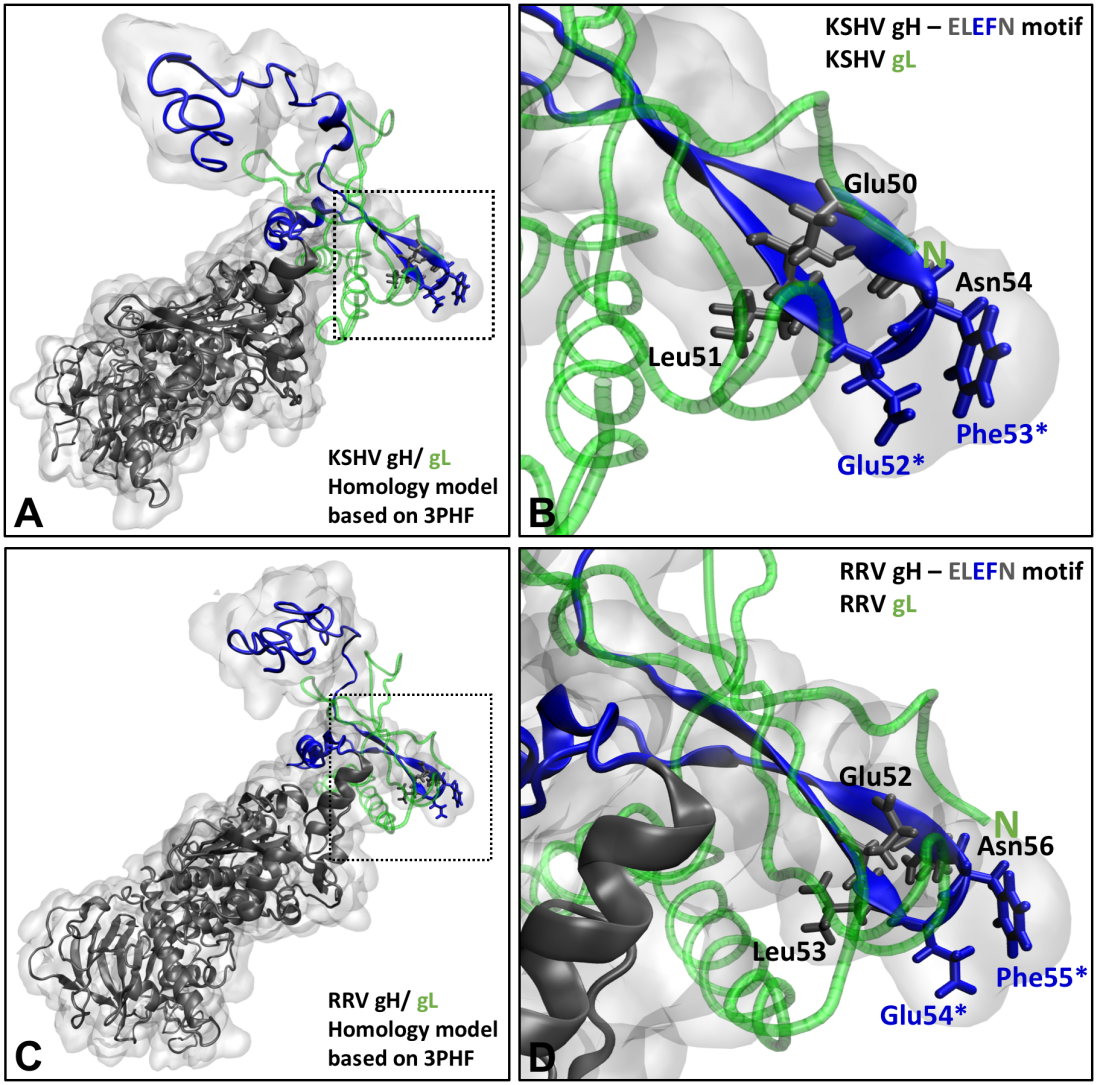
The E-L-E-F-N motif is located in a putative beta-hairpin at the KSHV and RRV gH/gL interaction site. Homology-based structure prediction of the KSHV gH/gL and RRV gH/gL complexes based on the crystal structure of the EBV gH/gL complex (PDB number 3PHF) using the Iterative Threading ASSembly Refinement (I-TASSER) server and the CO-THreader algorithms for protein-protein complex structure and multi-chain protein threading. **A** KSHV gH/gL complex. Domain I is colored in blue. gL is colored in green. **B** Enlarged view of the inset indicated in A by dotted lines, showing the E-L-E-F-N motif in a putative beta-hairpin. Amino acids Glu52 and Phe53 that are critical for Eph binding are highlighted by asterisks. **C** RRV gH/gL complex. Domain I is colored in blue. gL is colored in green. **D** Enlarged view of the inset indicated in C by dotted lines, showing the E-L-E-F-N motif in a putative beta-hairpin. Amino acids Glu54 and Phe55 that are critical for Eph binding are highlighted by asterisks.

The importance of the E-L-E-F-N motif and its surrounding region for binding of KSHV and RRV gH/gL to their respective high-affinity binding partners from the Eph family was additionally supported by a structural prediction of the KSHV and RRV gH/gL complex based on the crystal structure of the Epstein-Barr-Virus gH/gL complex [22]. In this prediction, the residues of gH crucial for Eph interaction *in vitro*, E52/F53 (KSHV) and E54/F55 (RRV), form the turn region residues of a putative beta-hairpin in an optimal array for protein-protein interaction (Fig 3). The spatial layout of gH and gL in this region suggests a possible stabilizing effect of the N-terminal domain of gL on the parallel beta-sheet structure. Formation of such a putative receptor-binding sub-domain of gH/gL fits with the observation that the N-terminal domain of gL is crucial for Eph interaction as well, as shown by co-immunoprecipitation of KSHV and RRV chimeras (Fig 1B).

### Viruses bearing mutations in the E-L-E-F-N motif are detargeted from Eph family receptors

**Fig 4:**
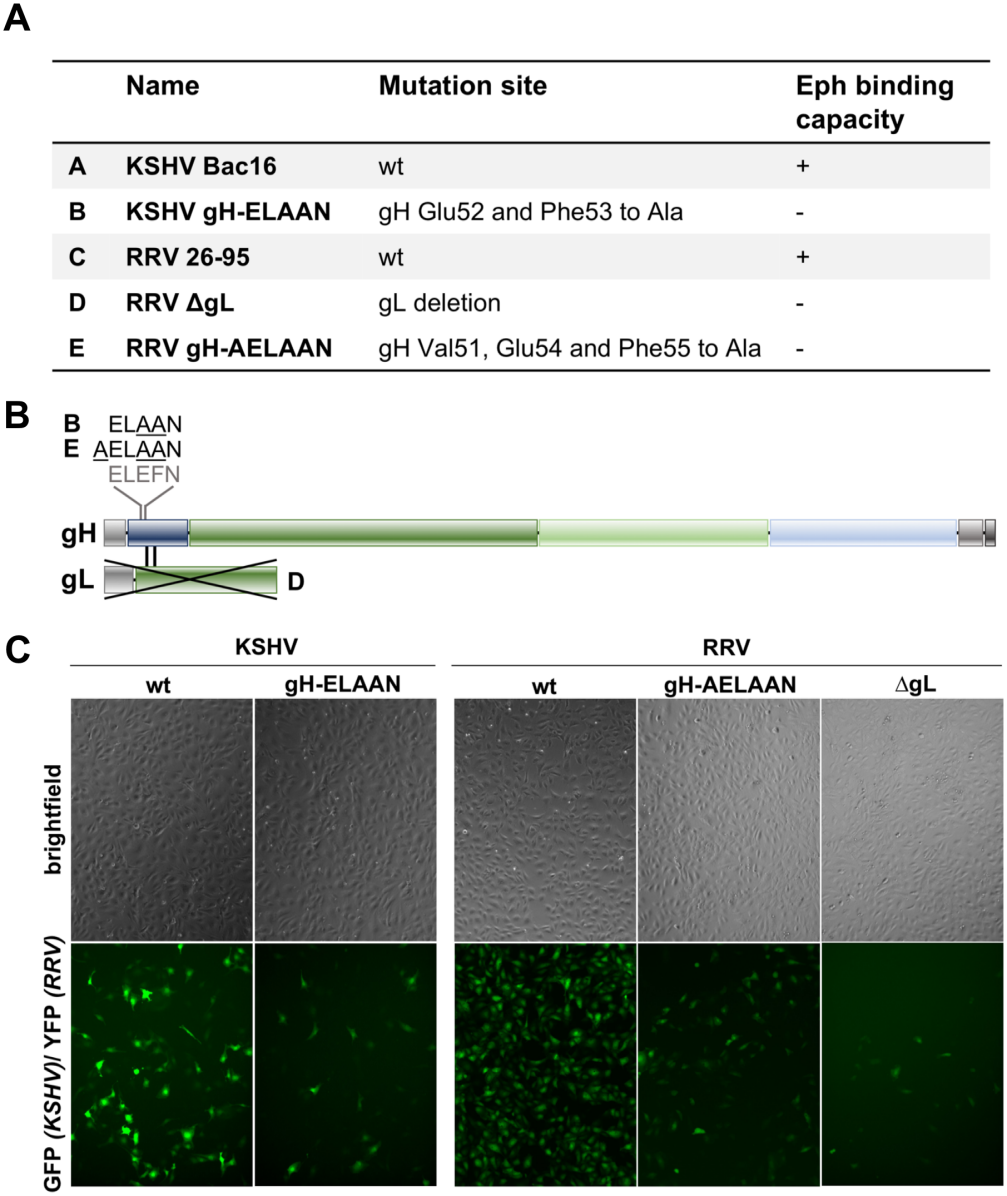
KSHV and RRV mutants. **A** List of bacmid-derived recombinant viruses generated for this study. **B** Schematic representation of mutations introduced into gH/gL. **C** SLK cells infected with wt or mutant KSHV or RRV.

Based on the results of our alanine scan and the structural predictions, we constructed mutant KSHV Bac16 and RRV-YFP 26–95, in which amino acids E52/F53 or E54/F55, respectively, are mutated to alanine (termed KSHV gH-ELAAN and RRV gH-AELAAN) (Fig 4A and 4B). For RRV we additionally mutated the valine at position 51 (V51) to alanine to avoid reversion as production of RRV stocks requires several rounds of lytic replication, which will select for revertants if these have a growth advantage. KSHV, on the other hand, is produced by induction of one lytic cycle after expansion of latently infected producer cells making emergence of revertants less likely. As an additional Eph-binding-deficient control we included a RRV-YFP 26–95 gL deletion mutant (RRV ΔgL) (Fig 4A and 4B). Western Blot analysis of KSHV and RRV wt and mutant virus particles verified that KSHV gH-ELAAN (S1D Fig) and RRV gH-AELAAN (S1E Fig) incorporate gH to wt levels and confirmed the complete deletion of gL on protein level for RRV ΔgL and the efficient incorporation of gL by RRV gH-AELAAN (S1E Fig). All gH mutants that were introduced into KSHV and RRV also reached expression levels comparable to wt when expressed alone or together with gL in transfected cells (S1C Fig). Analysis of KSHV gL in virus particles was precluded by our inability to generate antibodies to KSHV gL despite several attempts. All mutants were viable and infectious *in vitro* as assayed by the expression of green fluorescent protein (GFP) for KSHV or yellow fluorescent protein (YFP) for RRV under the control of a constitutively active promoter (Fig 4C).

**Fig 5:**
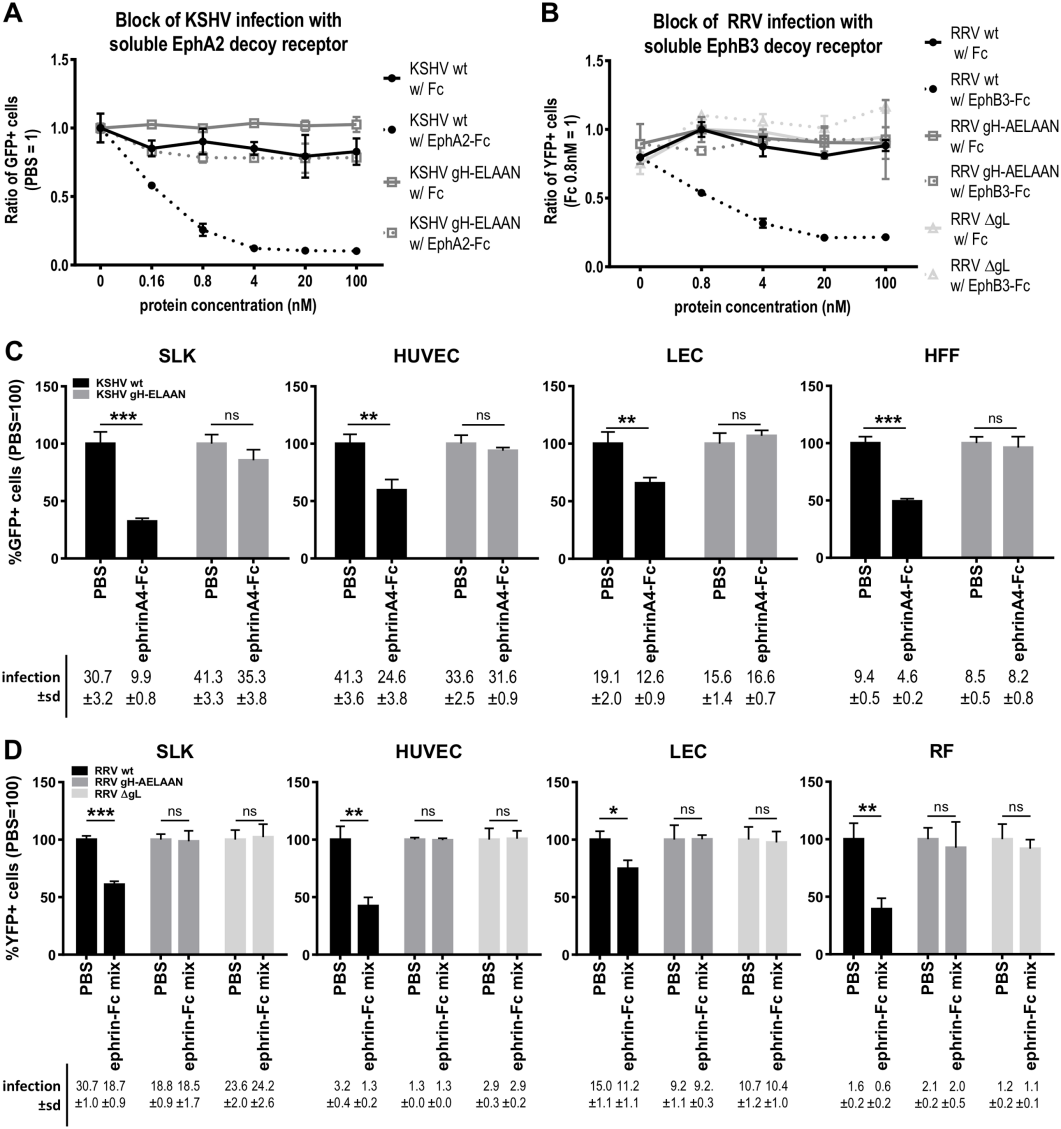
Mutation of the E-L-E-F-N motif is sufficient for Eph receptor detargeting. **A** Dose-dependent inhibition of KSHV infection by soluble EphA2-Fc on SLK cells. KSHV wt or gH-ELAAN were pre-incubated with EphA2-Fc. Fc alone and PBS were used as controls. GFP expression as indicator of infection was measured by flow cytometry. Infection without protein (PBS control) was set to 1 (duplicates, error bars represent range). **B** Dose-dependent inhibition of RRV infection by soluble EphB3-Fc on SLK cells. RRV 26–95 wt, ΔgL or gH-AELAAN were pre-incubated with EphB3-Fc. Fc alone and PBS were used as controls. YFP expression as indicator of infection was measured by flow cytometry. Infection with Fc 0.8nM was set to 1 (duplicates, error bars represent range). **C** Target cells were pre-incubated with a soluble ephrinA4-Fc fusion protein at 2μg/ml for 30min prior to infection with KSHV wt or gH-ELAAN. Infection was measured as in A. Infection without protein (PBS control) was set to 100% (triplicates, error bars represent sd). Non-normalized infection (%GFP+ cells) ±sd is listed below the respective bars. **D** Target cells were pre-incubated with a soluble ephrin-Fc fusion protein mix (ephrinA1, ephrinA2, ephrinA3, ephrinA4, ephrinA5, ephrinB1, ephrinB2, ephrinB3) at 2μg/ml each for 30min prior to infection with RRV 26–95 wt, ΔgL or gH-AELAAN. Infection was measured as in B. Infection without protein (PBS control) was set to 100% (triplicates, error bars represent sd). Non-normalized infection (%YFP+ cells) ±sd is listed below the respective bars. ns: not significant, *: p-value < 0.05, **: p-value < 0.01, ***: p-value < 0.001.

To evaluate the receptor usage of the described KSHV and RRV mutants we conducted blocking experiments by either pre-incubation of viral inocula with soluble Eph decoy receptor fused to the Fc part of IgG (EphA2-Fc/EphB3-Fc) (Fig 5A and 5B) or ligand competition on target cells to block access to Eph receptors (Fig 5C and 5D). For ligand-dependent blocking experiments of KSHV infection, we used recombinant ephrinA4 (ephrinA4-Fc) as a high affinity ligand [16,20], which blocks EphA2 on target cells. As RRV was shown to interact with both A- and B-type Ephs, a mix of all described recombinant ligands of Ephs (ephrinA1, ephrinA2, ephrinA3, ephrinA4, ephrinA5, ephrinB1, ephrinB2 and ephrinB3, each fused to Fc at the end of the extracellular part of the protein) was used in ligand competition experiments. For both viruses, wt and Eph-binding-negative mutants were titrated and normalized to achieve comparable infections in the absence of inhibitor.

In blocking experiments targeting viral particles, pre-incubation of the virus with soluble Fc alone as a control did not appreciably influence KSHV or RRV infection while soluble Eph decoy receptors led to a dose-dependent inhibition of KSHV infection of up to 90% (Fig 5A) and RRV infection of approximately 80% (Fig 5B) on SLK cells, as described before [15,16]. Contrarily, we observed no influence of saturating concentrations of EphA2-Fc/EphB3-Fc on the infection with Eph-binding-negative mutants KSHV gH-ELAAN, RRV gH-AELAAN or RRV ΔgL when compared to infection with untreated or soluble Fc treated viral inocula (Fig 5A and 5B). Correspondingly, soluble ephrinA4-Fc significantly reduced KSHV infection of SLK, human umbilical vein endothelial cells (HUVEC), lymphatic endothelial cells (LEC), and human foreskin fibroblasts (HFF) in a range from approximately 35% to 70% depending on the cell type (Fig 5C). Ligand competition using the recombinant ephrin-Fc mix resulted in similar, significant blocking of RRV infection of SLK, HUVEC, LEC, and rhesus monkey fibroblasts (RF) (Fig 5D). Pre-treatment of target cells with soluble ephrins did not influence infection with Eph receptor-detargeted virus mutants (Fig 5C and 5D), comparable to soluble decoy receptor pre-incubation.

In summary, using either soluble Eph decoy receptors or recombinant ephrins as blocking agents we observed a robust inhibition of infection with wt KSHV and RRV, while infection with Eph-detargeted mutants remained unaffected.

### Disruption of the Eph-binding motif does not affect virus attachment but results in reduced infectivity

**Fig 6:**
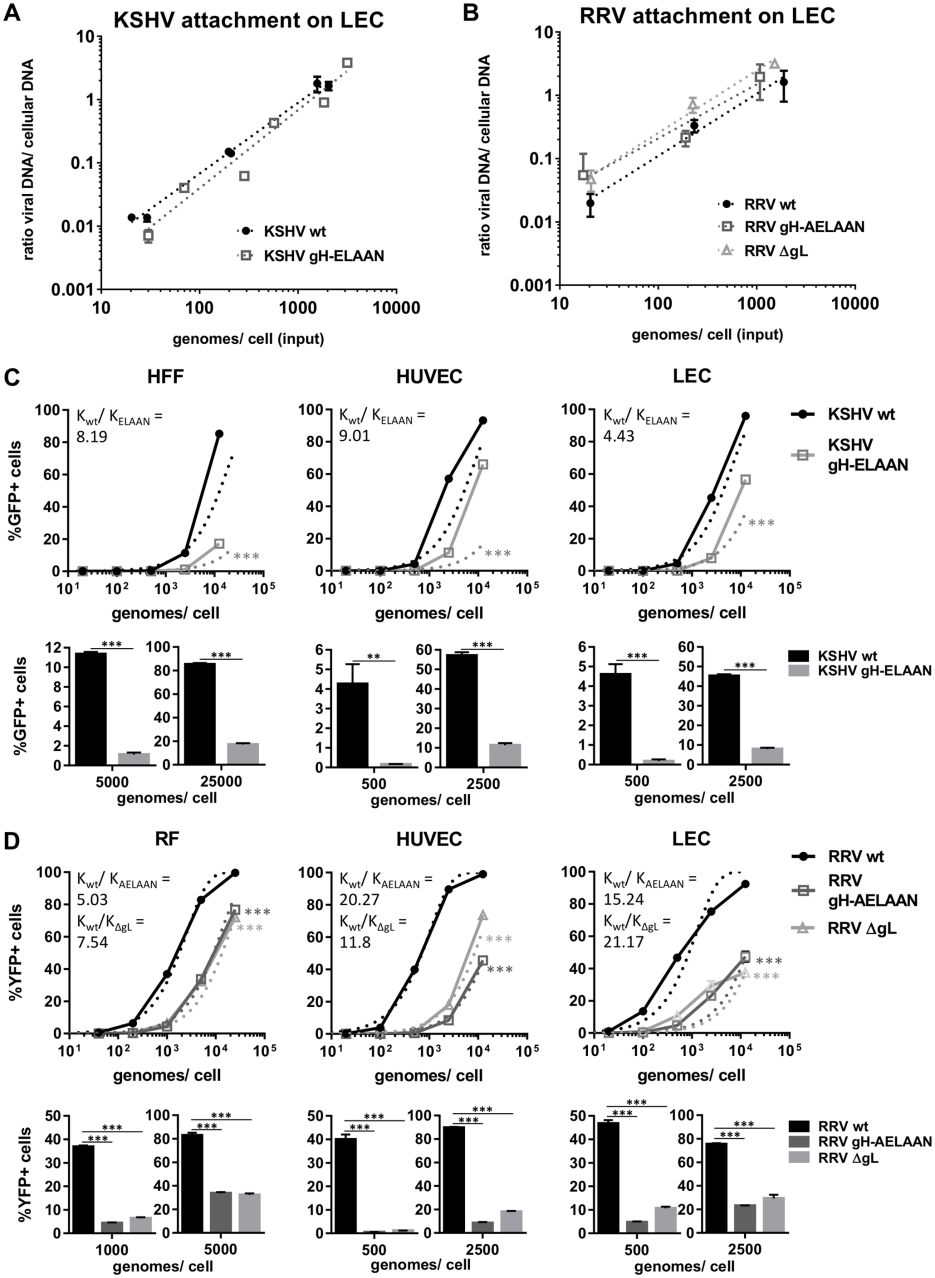
Eph-binding-negative RRV and KSHV mutants exhibit normal attachment and reduced specific infectivity. **A** Attachment of KSHV on LECs is not affected by mutational ablation of the Eph interaction. Cells were incubated with cold virus at the indicated concentrations at 4°C for 30min followed by genomic DNA isolation. The ratio of viral to cellular DNA as a measurement for attached virus was calculated based on ΔCt values of a genomic (CCR5) and a viral locus (ORF59, KSHV or ORF73, RRV) as determined by qPCR and plotted against input viral genome number. **B** Attachment of RRV on LECs is not affected by mutational ablation of the Eph interaction. Attachment was determined as in A. **C-D** Eph-binding-negative RRV and KSHV mutants exhibit a reduced specific infection. Target cells were infected with KSHV wt and gH-ELAAN (**C**) or RRV wt, gH-AELAAN and ΔgL (**D**) at the indicated virus concentrations. GFP (KSHV) or YFP (RRV) expression as indicator of infection was measured by flow cytometry. Solid lines represent one representive experiment (triplicates, error bars indicate sd). Dotted lines represent non-linear fitting of combined representative experiments of three independent pairs (KSHV) or two independent triplets (RRV) of virus stocks. The ratio K_wt_/K_mutant_ of the rate constant K of fitted curves for wt (K_wt_) and mutant viruses (K_ELAAN_, K_AELAAN_, K_ΔgL_) represents the effect of introduced mutations on specific infectivity. Bar graphs represent infections achieved by two specific input virus concentrations normalized to genome copies for one representative experiment per cell type. *: p-value < 0.05, **: p-value < 0.01, ***: p-value < 0.001.

To analyze the importance of the Eph interaction for cellular attachment and infectivity of KSHV and RRV particles, we normalized infectious dose to genome copies per cell. First the capacity of wt and mutant virus to bind target cells was analyzed. A comparison of the ratio of input genome copy numbers and bound genome copy numbers at 4°C revealed no differences in the attachment of KSHV wt and KSHV gH-ELAAN (Fig 6A) or RRV wt and RRV gH-AELAAN and RRV ΔgL (Fig 6B). We observed attachment comparable to wt of both Eph-binding-negative KSHV and RRV mutants over a range of 3 logs of input virus per cell (Fig 6A and 6B).

In contrast, KSHV gH-ELAAN and RRV gH-AELAAN/ΔgL exhibited a reduced specific infectivity on cells of epithelial (S2A and S2B Fig) and endothelial origin, as well as fibroblasts when compared to their corresponding wt virus. For both viruses, a representative experiment and averaged, fitted curves from repeat experiments are shown (Fig 6C and 6D, upper panels). The effect of the introduced mutations on specific infectivity was determined by the ratio of the rate constant K of fitted curves for wt and mutant viruses. K_wt_/K_mutant_ describes the shift of fitted curves of Eph-binding-negative viruses to the right indicating the fold increase in input virus required to achieve wt infection levels. The impairment of specific infectivity of KSHV gH-ELAAN ranged from a factor of 4.4 on LEC to a factor of 9 on HUVEC. Eph-binding-negative RRV mutants exhibited a reduction in specific infectivity from 5-fold for RRV gH-AELAAN on RF to approx. 20-fold for RRV gH-AELAAN and RRV ΔgL on HUVEC and LEC, respectively. Similarly, when comparing infection with an identical number of viral input genomes for wt and mutant KSHV and RRV, we observed a robust reduction in the percentage of infected cells with Eph-binding-negative viruses. For example, infection with KSHV was reduced approx. 5-fold to 10-fold on HFF and 5-fold to 25-fold on HUVEC or LEC when identical input genome numbers for KSHV wt and KSHV gH-ELAAN were compared in a range of 500 to 25000 genomes/cell (Fig 6C, bar graphs). Similarly, for RRV the reduction in the percentage of infected cells ranged from approx. 2.5-fold to 8.5-fold on RF and 2.6-fold to 10-fold on LEC to approx. 5-fold to 82-fold on HUVEC when comparing identical input genome numbers of wt and mutant viruses in a range of 500 to 5000 genomes/cell (Fig 6D, bar graphs). Results using the mean fluorescence intensity (MFI) of the reporter gene instead of percentage of infected cells as a readout corroborated the same conclusions (S2C and S2D Fig). Notably, RRV gH-AELAAN and RRV ΔgL performed highly similar in these assays.

#### Contribution of the gH/gL-Eph interaction to RRV infection *in vitro* is cell type-specific

As mentioned above, the extent of impairment of infection with RRV Eph-binding-negative viruses differed notably between analyzed cell types (Fig 6D). To explore this finding in more detail, we directly compared KSHV and RRV infection of wild type and mutant viruses on fibroblasts of human or rhesus monkey origin to primary human endothelial cells, plotting infection of fibroblasts against infection of endothelial cells for identical viral inocula.

Comparing KSHV and the Eph-binding-negative KSHV gH-ELAAN mutant on HFF and on LEC or HUVEC (S3 Fig), high variability was observed, which may be caused by fluctuating expression levels of e.g. Ephs or other factors in these primary cells as described before for HUVEC [15] and precludes a conclusion regarding the cell type-specific importance of Eph receptor usage for KSHV infection. Even though differences between some cell populations seem to exist, these were not consistently observed for KSHV.

**Fig 7:**
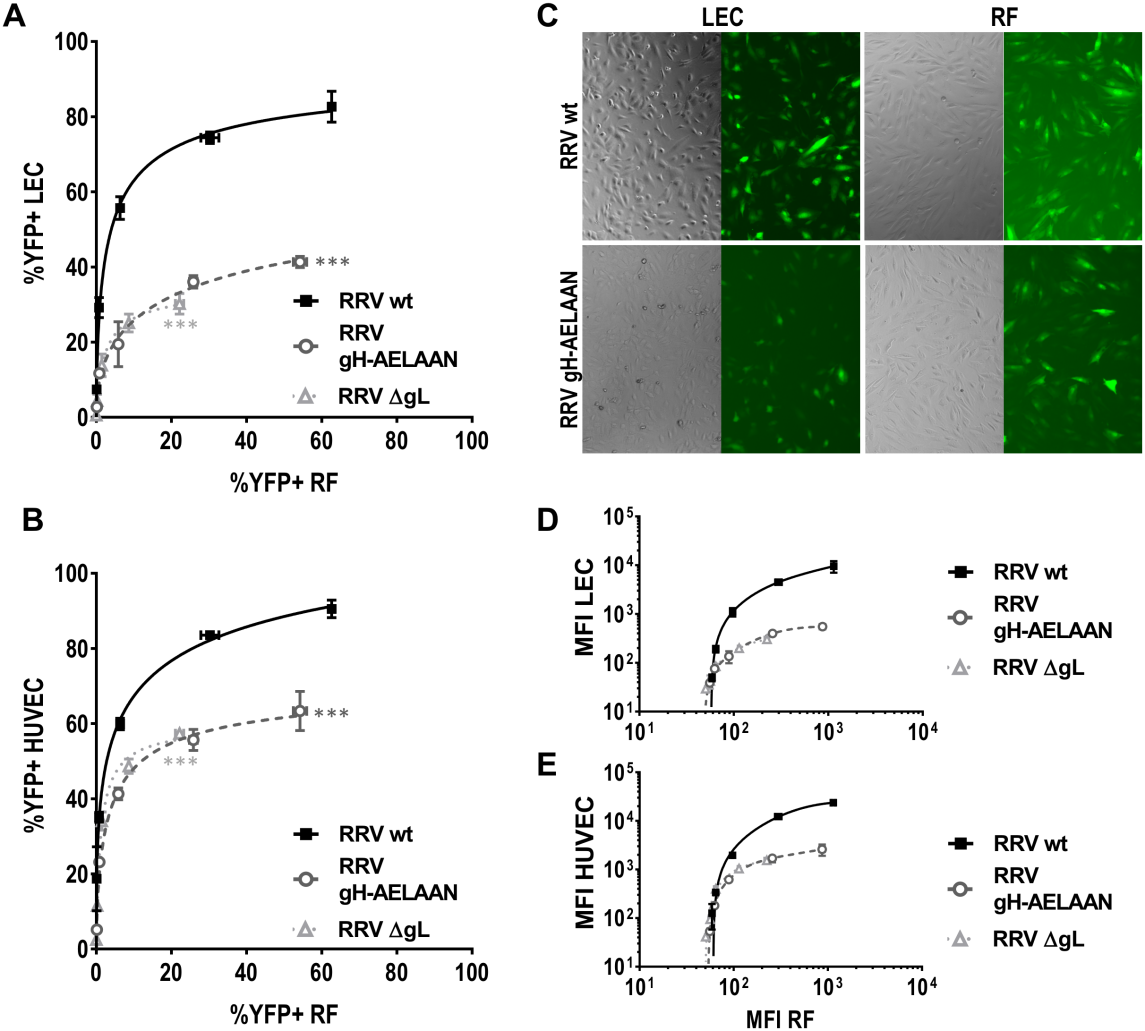
Contribution of the gH/gL-Eph interaction to RRV infection is cell type-specific. **A-B** Comparative infection on LEC **(A)** or HUVEC **(B)** and RF by RRV wt, RRV gH-AELAAN, and RRV ΔgL. RF and LEC or HUVEC were infected with the same inocula of the respective virus stock, and the percentage of reporter gene-positive cells as determined by flow cytometry for each dilution was plotted. **C** Micrograph of RF and LEC infected with the same respective inocula of wt and Eph-binding-negative RRV gH-AELAAN. **D-E** Comparative infection on LEC **(D)** or HUVEC **(E)** and RF by RRV wt, RRV gH-AELAAN, and RRV ΔgL as in A based on mean fluorescence intensity (MFI) as determined by flow cytometry. ***: p-value < 0.001.

For RRV on the other hand, the results followed a clear pattern. In all experiments (three out of three for all mutants on LEC, five out of five for RRV ΔgL on HUVEC, and four out of four for RRV gH-AELAAN on HUVEC each compared to RF) the Eph-binding-negative RRV mutants exhibited a stronger impairment on endothelial cells compared to fibroblasts (Fig 7). In a representative experiment, the same RRV wt inoculum that resulted in infection of approximately 10% on RF resulted in approx. 60% infection on LEC, whereas inocula of RRV gH-AELAAN and RRV ΔgL that resulted in approx. 10% infection on RF, yielded only around 25% infection on LEC (Fig 7A). Additionally, while from the second highest to the highest concentration, representing a twofold increase in input virus, infection of RF with RRV gH-AELAAN doubled, infection of LEC increased only marginally from approx. 35% to approx. 40% infected cells. Similarly, although to a lesser degree, we observed this phenotype when analyzing HUVECs as an alternative endothelial cell model (Fig 7B).

Using the MFI of the virally encoded reporter genes as a readout for infection paralleled results using the percentage of GFP+/YFP+ cells as a readout for infection with both KSHV and RRV (Fig 7D and 7E and S3 Fig). For RRV, using the MFI, the cell type-specific differences in infectivity amounted to about one log order of magnitude with regard to reporter gene intensity. Notably, results with RRV gH-AELAAN and RRV ΔgL vs RRV wt were again practically indistinguishable. As opposed to KSHV, this pattern was stably observed for different virus stocks as wells as different cell passages and batches of primary cells. Taken together, these results confirm that use of Eph family receptors plays a larger role for infection of endothelial cells than for infection of fibroblasts by RRV.

## Discussion

Eph family receptors, and specifically EphA2, have been described as host factors for a wide range of pathogens besides KSHV and RRV [15,16], including hepatitis C virus [32], Chlamydia trachomatis [33], Cryptococcus neoformans [34], malaria parasites [35], and as very recently reported EBV [36,37]. While it has been shown that KSHV interacts specifically with the ligand-binding domain of EphA2 in a manner that competes with the natural ephrin ligands [20], little was known until now about the specific interaction sites or motifs on the surface proteins of the respective pathogens. In this study, we present evidence for a conserved, distinctive binding site on DI of the gH/gL complex of the rhadinoviruses KSHV and RRV that is crucial for interaction with members of the Eph family of receptor tyrosine kinases.

Using a combination of comparative sequence and structural analysis together with *in vitro* mutational screens, we were able to map the Eph interaction site to the central amino acids of a conserved five amino acid motif Glu(E)-Leu(L)-Glu(E)-Phe(F)-Asn(N) on KSHV and RRV gH. Even though the amino acid sequence of gH is relatively variable within the herpesvirus family, and even so between KSHV and the RRV isolates 26–95 and 17577 (Fig 2A) as well as within a large number of RRV gH sequences isolated by Shin et al [38], the described E-L-E-F-N motif is strictly conserved in all KSHV and RRV gH sequences.

Direct evidence for the functional role of the conserved rhadinoviral Eph receptor interaction motif, and in particular of residues E52/F53 (KSHV) and E54/F55 (RRV) of gH, for Eph targeting was provided by the construction of KSHV gH-ELAAN and RRV gH-AELAAN virus mutants and subsequent inhibition experiments using either soluble decoy receptors (Fig 5A and 5B) or soluble ephrins (Fig 5C and 5D).

Interestingly, the conserved asparagine of the E-L-E-F-N motif is also a conserved N-glycosylation site, and its mutation leads to a visible shift in molecular weight (Fig 2B and 2C). While the asparagine itself does not seem to contribute to Eph binding, glycosylation could potentially play a role in steric shielding of this region from antibodies, as described for example for human immunodeficiency virus type 1 (HIV-1) gp120 [39], Ebola Virus (EBV) glycoprotein [40] and influenza virus hemagglutinin [41], as well as from MHC presentation [42].

High conservation on the amino acid sequence level also translates into an equally conserved structural prediction of the gH/gL complex of KSHV and RRV 26–95 (Fig 3), when modeled using the crystal structure of the EBV gH/gL complex [22]. In these computational models the amino acid residues E52/F53 of KSHV gH and E54/F55 of RRV gH that are crucial for Eph interaction are located in a predicted outward-angled beta-hairpin. Several reports indicate that the interaction of both A- and B-type ephrins with different Eph receptors is structurally conserved and mediated by insertion of the so-called G-H loop of ephrins into a conserved hydrophobic groove on the Eph receptors [31,43–45]. KSHV gH/gL interacts with the ephrin binding region of EphA2, which suggests that binding of rhadinoviral gH/gL complexes to Ephs may occur in a fashion that structurally mimics the binding of ephrins to their receptors. The importance of this structural motif is further supported by the effect of gH point mutants R59A, Y60A (RRV) and L60A, W62A (KSHV) on binding of Eph receptors but not on gH/gL complexation. In our model, these residues are located in the base region of the anti-parallel beta-sheets that form the beta-hairpin. Disruption of the base region may lead to a destabilization of the beta-hairpin structure and therefore loss of Eph receptor interaction on gH while binding to gL is maintained. Alternatively, these residues might directly contact Eph receptors and help determine the specificity of the interaction for e.g. EphA2 or EphB3.

According to our model, the putative beta-hairpin makes considerable contact with the N-terminal region of gL, suggesting that proper folding of the Eph interacting sub-domain may be gL-dependent. The importance of the N-terminal region of gL is also supported by our co-expression and immunoprecipitation experiments, in which the N-terminal regions of both gH and gL were necessary for Eph binding (Fig 1A and 1B). Furthermore, mutation of several amino acids adjacent to the E-L-E-F-N motif which led to a reduced gL interaction also led to reduced binding to EphA2 or EphB3, respectively, most likely due to an instability or a lack of gL in the gH/gL complex (Fig 2B and 2C). Our results are in good agreement with several reports on the functional importance of the N-terminal domain of the gH/gL complex for the entry process of EBV and VZV [21–23,46,47]. Our approach, however, does not exclude the existence of additional Eph interaction sites on gH/gL, which might determine the specificity of KSHV and RRV for individual Eph receptors. While this manuscript was in revision, EBV was reported to also interact with EphA2 not only through gH/gL, but additionally also through gB [36]. Whether the mechanism by which EBV and KSHV interact with EphA2 is conserved remains to be determined. Likely, differences do exist as EBV fuses directly with the membrane of epithelial cells, whereas KSHV enters through endocytosis. To validate our model and to fully elucidate structural aspects of the role of the rhadinoviral gH DI, as well as of potential additional interaction sites, for receptor binding and entry into target cells, crystal structures of rhadinoviral gH/gL complexes are needed, preferably in complex with the respective high affinity receptor from the Eph family.

Surprisingly, the deletion of *orf47*, which encodes gL, from the RRV genome did not abrogate the production of infectious virus particles. Similar attempts at deleting gL of KSHV were so far unsuccessful as recombinant bacmids harboring the corresponding deletion did not yield infectious virus. One explanation for this might be the function of recently described spliced genes enclosing the *orf47-orf46-orf45* locus during reactivation from latency [48]. RRV, however, replicates lytically on RFs and rarely enters latency, which could mask defects in reactivation and make potential homologous RRV transcripts dispensable for RRV growth in culture. Accordingly, in our experiments RRV ΔgL performed practically indistinguishable from RRV gH-AELAAN. Interestingly, an essential role for infectivity was described for gL of herpes simplex virus type 1 [49] and both human and rhesus cytomegalovirus [50], whereas gL was described as non-essential for infectivity and cell-to-cell spread of the murine gamma-herpesvirus MHV-68 [51]. Similarly, pseudorabiesvirus gL null mutants remain infectious, although exhibiting impaired entry and cell-to-cell spread [52–54]. Thus, whether gL is essential for infectivity *in vitro* or not seems to vary within the herpesviruses.

The question of the quantitative contribution of Eph receptors to rhadinovirus infection could not be answered satisfactorily until now, due to the intrinsic limitations of blocking assays, such as concentration and amount of blocking agent, and possible confounders of knockout experiments, such as potential use of alternative Eph receptors or detrimental effect of the knockout on the cell in general. The dramatic inhibition of KSHV and RRV wt infection demonstrated by specifically targeting the Eph interaction or after EphA2 knockdown or knockout [15,16,18,55] is similar in extent to what we observed using a soluble decoy receptor block (Fig 5A and 5B). This is also in very good agreement with our experiments analyzing the specific infectivity of our mutants where the percentage of infected cells with Eph-binding-deficient mutant virus was reduced 2.5-fold to 82-fold when compared to wt virus using equal input genome numbers. Similarly, approx. 4 to 20 times more Eph-binding-deficient mutant virus was needed to achieve infection levels comparable to those of wt virus (Fig 6C and 6D). Our results now quite unequivocally demonstrate that the interaction of gH/gL with Ephs is not essential for KSHV and RRV infection but contributes significantly to infectivity.

The interaction of gH with different viral or cellular proteins is thought to be a determinant of cell tropism in a wide range of herpesviruses [56–58,21]. In previous studies, we observed differences in the amount of inhibition of KSHV and RRV infection achieved by blocking the Eph interaction [15,16] depending on the cell type. While for KSHV, the cell type-specific defect in infectivity of KSHV gH-ELAAN varied significantly between different batches of fibroblasts and endothelial cells and did not allow for a clear conclusion, for RRV the cell type-specific differences were consistently observed. On LEC and HUVEC, both RRV gH-AELAAN and RRV ΔgL exhibited a defect in infectivity that was more pronounced than that observed on primary RF. These results confirm our previous findings that Eph receptors play a minor role in the infection of fibroblasts compared to infection of endothelial cells by RRV [16], as also exemplified by the ability of RRV gH-AELAAN and RRV ΔgL to replicate to high titers on RF.

The observation that ablation of the Eph interaction does not fully abrogate infectivity and has cell type-specific effects for RRV suggests the contribution of yet to be identified additional host factors for RRV and potentially also KSHV; for RRV in particular as integrins do not seem to play a major role for RRV [14]. This becomes most apparent for the infection of lympathic endothelial cells with Eph-binding-deficient RRV mutants. Despite increasing amounts of input virus the infection appeared to be approaching a plateau at around 50% infected cells (Figs 6D and 7A), which is in good agreement with previous observations where RRV failed to infect a sizable subpopulation of endothelial cells despite increasing amounts of input virus when the Eph interaction was blocked [16]. In our opinion, the most likely explanation would be that the cell population that is refractory to Eph-independent infection lacks a host factor that can functionally substitute for the Ephs.

The critical sub-domain on the gH/gL glycoprotein complex identified in our study may serve as a target for inhibition by monoclonal antibodies. We identified several critical amino acid residues that most likely mediate direct interaction with Eph receptors. Even if the gH/gL-Eph interaction is not strictly essential, strong inhibition of KSHV infection can be achieved by targeting this region. Blocking this highly conserved region with an antibody might afford inhibition similar to that achieved by soluble EphA2-Fc decoy receptor (Fig 5A), which presumably binds in a manner that should be very similar to antibodies targeting the E-L-E-F-N motif.

The Eph-ephrin system is a complicated signaling network that may play a role beyond simple receptor interaction in the viral infection and that interacts with a surprising number of pathogens. With the construction of Eph-binding-negative rhadinovirus mutants described in this paper we not only present direct evidence for a conserved Eph interaction motif of KSHV and RRV, but also provide a useful toolkit for the future analysis of EphA2-specific signaling in the case of KSHV and signaling of a broader range of both A-type and B-type Ephs for RRV.

## Materials and methods

### Cells

A549 [59] (laboratory of Stefan Pöhlmann, German Primate Center—Leibniz Institute for Primate Research, Göttingen, Germany), Human embryonic kidney (HEK) 293T cells [60,61] (laboratory of Stefan Pöhlmann), human foreskin fibroblasts (HFF) (laboratory of Klaus Korn, Universitätsklinikum Erlangen, Institute for Clinical and Molecular Virology, Erlangen, Germany), SLK cells [62,63] (NIH AIDS Research and Reference Reagent program) and rhesus monkey fibroblasts (RF) (laboratory of Prof. Rüdiger Beer, German Primate Center—Leibniz Institute for Primate Research, Göttingen, Germany) were cultured in Dulbecco’s Modified Eagle Medium (DMEM), high glucose, GlutaMAX, 25mM HEPES (Thermo Fisher Scientific) supplemented with 10% fetal calf serum (FCS) (Thermo Fisher Scientific), and 50μg/ml gentamycin (PAN Biotech). iSLK cells [64] (laboratory of Don Ganem, Novartis Institutes for BioMedical Research, Emeryville, CA, USA) were maintained in DMEM supplemented with 10% FCS, 50μg/ml gentamycin, 2.5μg/ml puromycin (InvivoGen) and 250μg/ml G418 (Carl Roth). Human vascular endothelial cells (HUVEC) (PromoCell) were maintained in standard Endothelial Cell Growth Medium 2 (PromoCell). Human lymphatic endothelial cells (LEC) from juvenile donors (a kind gift from Anja Boos, Universitätsklinikum Erlangen, Department of Plastic and Hand Surgery, Erlangen, Germany) were maintained in Endothelial Cell Growth Medium MV 2 (PromoCell).

### BAC mutagenesis, virus production, viral nucleic acid isolation and analysis

Eph-interaction-negative KSHV (KSHV gH-ELAAN) and RRV (RRV gH-AELAAN, RRV ΔgL) recombinants were generated using a two-step, markerless λ-red-mediated BAC recombination strategy as described by Tischer et al. [65]. KSHV gH-ELAAN and RRV gH-AELAAN harbor amino acid substitutions E52A and F53A (KSHV) or V51A, E54A and F55A respectively. The deletion in RRV ΔgL encompasses 128 nucleotides from position 78 to position 205 (amino acids 27 through 68), resulting in a frameshift after amino acid 26 and a stop codon after 37 amino acids. Deletion in this region was chosen in order not to destroy known and potential overlapping genes that may so far not have been charted, and because we identified sequences reminiscent of regulatory elements in the region directly surrounding the start codon. Bacmid clones BAC16 (KSHV) [66] and BAC35-8 (RRV) were used, respectively. In short, recombination cassettes were generated from the pEPKanS template by polymerase chain reaction (PCR) with Phusion High Fidelity DNA polymerase (Thermo Fisher Scientific) using long oligonucleotides (Ultramers; purchased from Integrated DNA Technologies (IDT)) (see S1 Table for a complete list of primers). Recombination cassettes were transformed into BAC16-carrying *Escherichia coli* strain GS1783 or RRV-YFP-carrying GS1783 respectively, followed by kanamycin selection, and subsequent second recombination under 1% L(+)arabinose (Sigma-Aldrich)-induced I-SceI expression. Colonies were verified by PCR of the mutated region followed by sequence analysis (Macrogen), pulsed-field gel electrophoresis and restriction fragment length polymorphism. For this purpose, bacmid DNA was isolated by standard alkaline lysis from 5ml liquid cultures. Subsequently, the integrity of bacmid DNA was analyzed by digestion with restriction enzyme *Xho*I and separation in 0.8% PFGE agarose (Bio-Rad) gels and 0.5×TBE buffer by pulsed-field gel electrophoresis at 6 V/cm, 120-degree field angle, switch time linearly ramped from 1s to 5s over 16 h (CHEF DR III, Bio-Rad). Infectious KSHV recombinants were generated by transfection of purified bacmid DNA (NucleoBond Xtra Midi (Macherey-Nagel)) into iSLK cells using GenJet Ver. II (Signagen) according to manufacturer’s instructions. After visible GFP expression, selection was performed using 200μg/ml hygromycin B (InvivoGen) until only GFP positive cells remained. Lytic replication of KSHV-BAC16 was induced in DMEM supplemented with 10% fetal calf serum (FCS) and 50μg/ml gentamycin by addition of 2.5mM sodium-butyrate and 1μg/ml doxycycline. Supernatant was harvested after the cell monolayer was destroyed.

For RRV, infectious recombinants were generated by transfection of purified bacmid DNA (NucleoBond Xtra Midi) into 293T cells with GenJet Ver. II (Signagen) according to manufacturer’s instructions. After 2 days, BAC-transfected 293T cells were transferred onto a confluent rhesus monkey fibroblasts monolayer and co-cultivated until a visible cytopathic effect (CPE) was observed. Virus stocks were prepared by inoculating fresh primary rhesus monkey fibroblasts with virus containing supernatant of 293T/rhesus monkey fibroblast co-cultures at a very low multiplicity of infection (MOI; about 1 infected cell in 1000 cells) and letting the virus replicate until the cell monolayer was destroyed. Virus-containing cell supernatant from iSLKs and rhesus monkey fibroblasts was clarified by centrifugation (4750g, 10 minutes), concentrated by overnight centrifugation (4200rpm, 4°C) and careful aspiration of approximately 95% of the supernatant. The pellet was resuspended overnight in the remaining liquid. Stocks of wt and recombinant viruses were aliquoted and stored at -80°C. The integrity of the L-DNA part of virus recombinants was confirmed by Illumina-based next-generation sequencing. For isolation of viral DNA, concentrated virus stocks were incubated with DNAseI (40 units/ml) for 1h at 37°C. Addition of EDTA to a final concentration of 15mM was followed by a second incubation step (70°C, 10min). After a third incubation step with ProteinaseK (1mg/ml) and SDS (0.5%) (60°C, 2h) standard phenol chloroform extraction was performed. Sample preparation was performed using the Nextera DNA Sample Preparation system, dual indexing, and sequencing using the MiSeq Reagent Kit, 600 Cycles on the Illumina MiSeq system. Demultiplexed paired 300 nt sequence reads were analyzed by Genomics Workbench 10 (Qiagen Bioinformatics, Aarhus, DK).

### Plasmids

pcDNA4 vectors containing full-length EphA2 (ref|NM_004431|, pcDNA4-EphA2-myc), the soluble ectodomain of EphA2 (amino acids 1–534) (ref|NM_004431|, pcDNA4-EphA2-HA), a soluble EphA2-Fc fusion construct comprising amino acids 25–534 of human EphA2 in the pAB61 Fc-fusion backbone vector (pEphA2-Fc) [15] and EphB3 (ref|BC052968|, pcDNA-EphB3-myc) [15,16] were described elsewhere. KSHV/RRV chimeric gH constructs were generated based on pcDNA6aV5His/pcDNA3.1 backbone vectors containing RRV/KSHV gH and gL coding sequences (ref|GQ994935.1|, pcDNA6aV5-KSHV-gH, pcDNA3.1-KSHV-gL-Flag; ref|AF210726.1|, pcDNA6aV5-RRV-gH, pcDNA3.1-RRV-gL-Flag) [15,16] by PCR based restriction enzyme cloning. The KSHV gLΔ135-164-Flag construct (pcDNA3.1-KSHV-gLΔ135-164-Flag) was generated by a PCR-based mutagenesis using phosphorylated primers followed by blunt end ligation of the PCR product (see S1 Table for a complete list of primers and constructs). Plasmids harboring point mutations in domain I of RRV/KSHV gH used in the alanine scan were purchased from GenScript based on pcDNA6aV5-KSHV-gH and pcDNA6aV5-RRV-gH.

### Recombinant proteins

Recombinant, soluble EphA2 Fc/Strep-fusion protein was purified under native conditions by Strep-Tactin chromatography from 293T cell culture supernatant. 293T cells were transfected by Polyethylenimine "Max" (PEI) (Polysciences) [67] transfection with pEphA2-Fc. The protein-containing cell culture supernatant was filtered through 0.22μm PES membranes (Millipore) and passed over 0.5ml of a Strep-Tactin Superflow (IBA Lifesciences) matrix in a gravity flow Omniprep column (BioRad). Bound protein was washed with approximately 50ml phosphate buffered saline pH 7.4 (PBS) and eluted in 1ml fractions with 3mM desthiobiotin (Sigma-Aldrich) in PBS. Protein-containing fractions were pooled and buffer exchange to PBS via VivaSpin columns (Sartorius) was performed. Protein concentration was determined by absorbance at 280nm. Aliquots were frozen and stored at −80°C. Recombinant, human, soluble EphB3-Fc (5667-B3-050) and soluble ephrin ligands, as either human (rh) or mouse (rm) Fc-fusion proteins (rm-ephrinA1, rm-ephrinA2, rh-ephrinA3, rh-ephrinA4, rh-ephrinA5, rm-ephrinB1, rm-ephrinB2 and rh-ephrinB3 Fc) (SMPK3) were purchased from R&D Systems.

### Quantitative realtime-PCR-based viral genome copy number analysis and virus attachment assay

Concentrated virus samples were treated with DNAseI (0.1 units/μl) to remove any non-encapsidated DNA (37°C, overnight). Subsequently, DNAseI was deactivated and viral capsids were disrupted by heating the samples to 95°C for 30 minutes. Realtime-PCR (qPCR) was performed on a StepOne Plus cycler (Thermo Fisher Scientific) in 20μl reactions using the SensiFAST Probe Hi-ROX Kit (Bioline) (cycling conditions: 3min initial denaturation at 95°C, 40 cycles 95°C for 10s and 60°C for 35s). All primer-probe sets were purchased from IDT as complete PrimeTime qPCR Assays (primer:probe ratio = 4:1). Samples were analyzed in technical triplicates. A series of six 10-fold dilutions of bacmid DNA was used as standard for absolute quantification of viral genome copies based on qPCR of ORF59 for KSHV and ORF73 for RRV (see S1 Table for a complete list of primers). For virus attachment assays LEC were plated at 50 000 cells/cm^2^. Target cells were incubated with ice-cold virus dilutions at the indicated concentrations, normalized to genomes per cell, at 4°C for 30min. After three washes with ice-cold PBS genomic DNA was isolated using the ISOLATE II Genomic DNA Kit (Bioline) according to manufacturer’s instructions. The ratio of viral DNA to cellular DNA as a measurement of attached virus was determined by qPCR as described above. Relative values of bound viral genomes to cellular DNA were calculated on the basis of ΔCt values for viral genomic loci (ORF59 for KSHV, ORF73 for RRV) and a cellular genomic locus (CCR5).

### Infection assays and flow cytometry

For infection assays cells were plated at 50 000 cells/cm^2^ (SLK, HUVEC, LEC) or 25 000 cells/cm^2^ (RF, HFF) respectively. One day after plating, cells were infected with the indicated amounts of virus. 24h or 48h post infection cells were harvested by brief trypsinization, followed by addition of 5% FCS in PBS to inhibit trypsin activity, spun down (1200rpm, 10min), washed once with PBS, re-pelleted and fixed in PBS supplemented with 2% formaldehyde (Carl Roth). A minimum of 10 000 cells was analyzed per sample for GFP or YFP expression on a LSRII flow cytometer (BD Biosciences). Data was analyzed using Flowing Software (Version 2.5). For block with soluble ephrins, cells were pre-incubated with the indicated ephrin-Fc fusion proteins at a final concentration of 2μg/ml for 30min at room temperature followed by infection with KSHV or RRV. Block of KSHV/RRV infection with soluble decoy receptor was assayed by infection with virus inocula that were pre-incubated with the indicated concentrations of soluble EphA2-Fc, EphB3-Fc or Fc alone at room temperature for 30min.

### Immunoprecipitation and Western blot analysis

293T cells were transfected using PEI [67] or Lipofectamine with Plus reagent (Thermo Fisher Scientific) as per the manufacturer’s instructions. Recombinant gH-V5/gL-Flag complexes were precipitated from the lysates of 293T cells transfected with the respective expression constructs. Lysates were prepared in NP40 lysis buffer (1% Nonidet P40 Substitute (Sigma-Aldrich), 150mM NaCl (Sigma-Aldrich), 50mM HEPES (VWR), 1mM EDTA (Amresco) with freshly added Protease Inhibitor Cocktail, General Use (Amresco)) and subsequently incubated with agitation with 0.5μg V5-tag antibody (Serotec or Bio-Rad) and ProteinG sepharose (GenScript or GE Healthcare) for 2h or overnight at 4°C. Amount of input gH/gL between mutants was normalized by diluting lysates with cell lysate from non-transfected 293T cells prior to immunoprecipitation according to Western blot analysis of lysates and evaluation of the gH/gL content for each construct. After one wash, pre-coupled complexes were incubated overnight at 4°C with agitation with equal amounts of lysate of full-length EphA2-myc or full-length EphB3-myc expression plasmid transfected 293T cells (for KSHV gH/gL or RRV gH/gL binding analysis, respectively) or with supernatant of 293T cells transfected with an expression plasmid for HA-tagged EphA2 ectodomain (for KSHV gH/gL binding analysis). ProteinG beads were collected by brief centrifugation and washed 3 times in NP40 lysis buffer. Precipitates were heated in SDS sample buffer and analyzed by polyacrylamide gel electrophoresis (PAGE) using 8–16% tris-glycine gradient gels (Thermo Fisher Scientific) and Western blot (100mA, max 30V, 1h in Towbin buffer (25mM Tris, 192mM glycine)). For Western blot analysis of virus particles, a 5% OptiPrep (Sigma-Aldrich) in PBS cushion was overlayed with 1ml concentrated virus stock and centrifuged for 2h at 20 000g. Approx. 95% of the supernatant was discarded and the virus pellet was washed once with 1ml PBS and spun down (20 000g, 1h). The virus pellet was resuspended in 30μl PBS, dissolved overnight and subsequently heated after the addition of 50μl SDS sample buffer (99°C, 15min). Western Blot analysis was performed as described above.

### Structure prediction and analysis

Homology based structure prediction was performed using the Iterative Threading ASSembly Refinement (I-TASSER) server on standard settings for structure prediction of KSHV or RRV gH based on the crystal structure of the EBV gH/gL complex (3PHF). Modeling of the KSHV or RRV gH/gL complexes was additionally performed using both the SPRING and CO-THreader algorithms for protein-protein complex structure and multi-chain protein threading with no differences between determined structures. Resulting CO-THreader and I-TASSER structures were aligned with the VMD 1.9.3 OpenGL RMSD Trajectory Tool based on the overlapping region of gH domain I predicted in both models (KSHV amino acids 43 to 87, RRV amino acids 45 to 88) at an RMSD of 0.589Å for KSHV and 0.616Å for RRV. All further analyses and visualizations were performed using VMD 1.9.3 OpenGL.

### Mathematical and statistical analysis

Curve fitting of specific infectivity normalized to genome copies/cell was performed using the built-in exponential equation for one phase association of GraphPad Prism version 6 for Windows (GraphPad Software, La Jolla California USA) based on the poisson distribution [68]. The span was set from 0 to 100, representing 0% or 100% infected cells, respectively, resulting in the simplified function f(x) = 100*(1-*e*^-K^*^x^), with x representing input genome number and K representing the specific infectivity per input genome. The ratio between K_wt_ and K_mutant_ was used to calculate the differences in infectivity between wt and mutant viruses. Statistical difference between fitted curves was determined by the extra sum-of-squares F test with confidence intervals corrected for multiple comparisons using the Bonferroni correction. Statistical analysis of multiple groups was performed using regular two-way analysis of variance (ANOVA) followed by Sidak’s multiple comparison test. Statistical difference between two groups was determined by unpaired Student’s t-tests followed by correction for multiple comparison using the Holm-Sidak method when necessary. All Statistical analyses were performed with GraphPad Prism version 6. For all statistics, *: p-value < 0.05, **: p-value < 0.01, ***: p-value < 0.001.

### Primers

See S1 Table for a complete list of primers.

### Antibodies

See S1 Table for a complete list of antibodies.

## Supporting information

S1 Fig Effect of single point mutations in the N-terminal domain of gH on gH/gL stability and complexation as well as gH and gL incorporation in the virus particles. **A** Effects of point mutations on the stability of KSHV gH/gL complexes in the absence of recombinant Eph receptors. V5-tagged KSHV gH mutants were co-expressed with Flag-tagged KSHV gL. gH-V5/gL-Flag complexes were immunoprecipitated using monoclonal antibody to the V5-tag and precipitates were analyzed by Western blot. KSHV gH/RRV gL and KSHV gH alone serve as negative control. **B** Effects of point mutations on the stability of RRV gH/gL complexes in the absence of recombinant Eph receptors. V5-tagged RRV gH mutants were co-expressed with Flag-tagged RRV gL. gH-V5/gL-Flag complexes were immunoprecipitated and analyzed as in A. KSHV gH/RRV gL and RRV gH alone serve as negative control. **C** Point mutations in the E-L-E-F-N motif of KSHV and RRV gH do not influence stability of gH alone or of the gH/gL complex. V5-tagged KSHV gH wt, gH E52AF53A (gH-ELAAN), RRV gH wt and gH V51AE54AF55A (gH-AELAAN) were either expressed alone or co-expressed with Flag-tagged KSHV/RRV gL. gH-V5 and gH-V5/gL-Flag complexes were immunoprecipitated and analyzed as in A. **D** Double mutation E52AF53A in KSHV gH does not influence the incorporation of gH into the virus particle. KSHV wt, and gH-ELAAN virus preparations were analyzed by Western Blot. K8.1 was used as loading control. K8.1 runs in a diffuse molecular weight pattern due to its complex O-glycosylation. **E** Triple mutation V51AE54AF55A in RRV gH (gH-AELAAN) does not influence the incorporation of gH and gL into the virus particle. RRV wt, gH-AELAAN and RRV ΔgL virus preparations were analyzed by Western Blot. gB was used as loading control. Abbreviations: IP: immunoprecipitation, IB: immunoblotting.
(TIF)

S2 Fig Specific infectivity of Eph-binding-negative RRV and KSHV mutants. **A-B** Eph-binding-negative RRV and KSHV mutants exhibit a reduced specific infection on epithelial cells. Target cells were infected with KSHV wt and gH-ELAAN (**A**) or RRV wt, gH-AELAAN and ΔgL (**B**) at the indicated virus concentrations. GFP (KSHV) or YFP (RRV) expression as indicator of infection was measured by flow cytometry (triplicates, error bars indicate sd). **C-D** Eph-binding-negative RRV and KSHV mutants exhibit a reduced specific infection assayed by mean fluorescence intensity (MFI) of the respective reporter gene. Target cells were infected with KSHV wt and gH-ELAAN (**C**) or RRV wt, gH-AELAAN and ΔgL (**D**) at the indicated virus concentrations. GFP (KSHV) or YFP (RRV) MFI as indicator of infection was measured by flow cytometry (triplicates, error bars indicate sd).
(TIF)

S3 Fig Contribution of the gH/gL-Eph interaction to KSHV infection of endothelial cells and fibroblasts. **A-B** Comparison of KSHV wt with KSHV gH-ELAAN infection based on GFP reporter gene-positive cells on LEC **(A)** or HUVEC **(B)** and HFF. HFF and LEC or HUVEC were infected with the same inocula of the respective virus stock, and the percentage of reporter gene-positive cells as determined by flow cytometry for each dilution was plotted. **C** Micrograph of HFF and LEC infected with the same inocula of wt and Eph-binding-negative KSHV. **D-E** Comparison of KSHV wt and KSHV gH-ELAAN infection based on MFI on LEC **(D)** or HUVEC **(E)** and HFF performed as in (A-B).
(TIF)

S1 Table List of accession numbers, primers, and antibodies used in this study. (XLSX)
