# Supplementary figures and images for: A conserved Eph family receptor-binding motif on the gH/gL complex of Kaposi’s sarcoma-associated herpesvirus and rhesus monkey rhadinovirus
Source: PLoS Pathog. 2018 Feb 12;14(2):e1006912. doi: 10.1371/journal.ppat.1006912 (PMC5825162; doi:10.1371/journal.ppat.1006912)

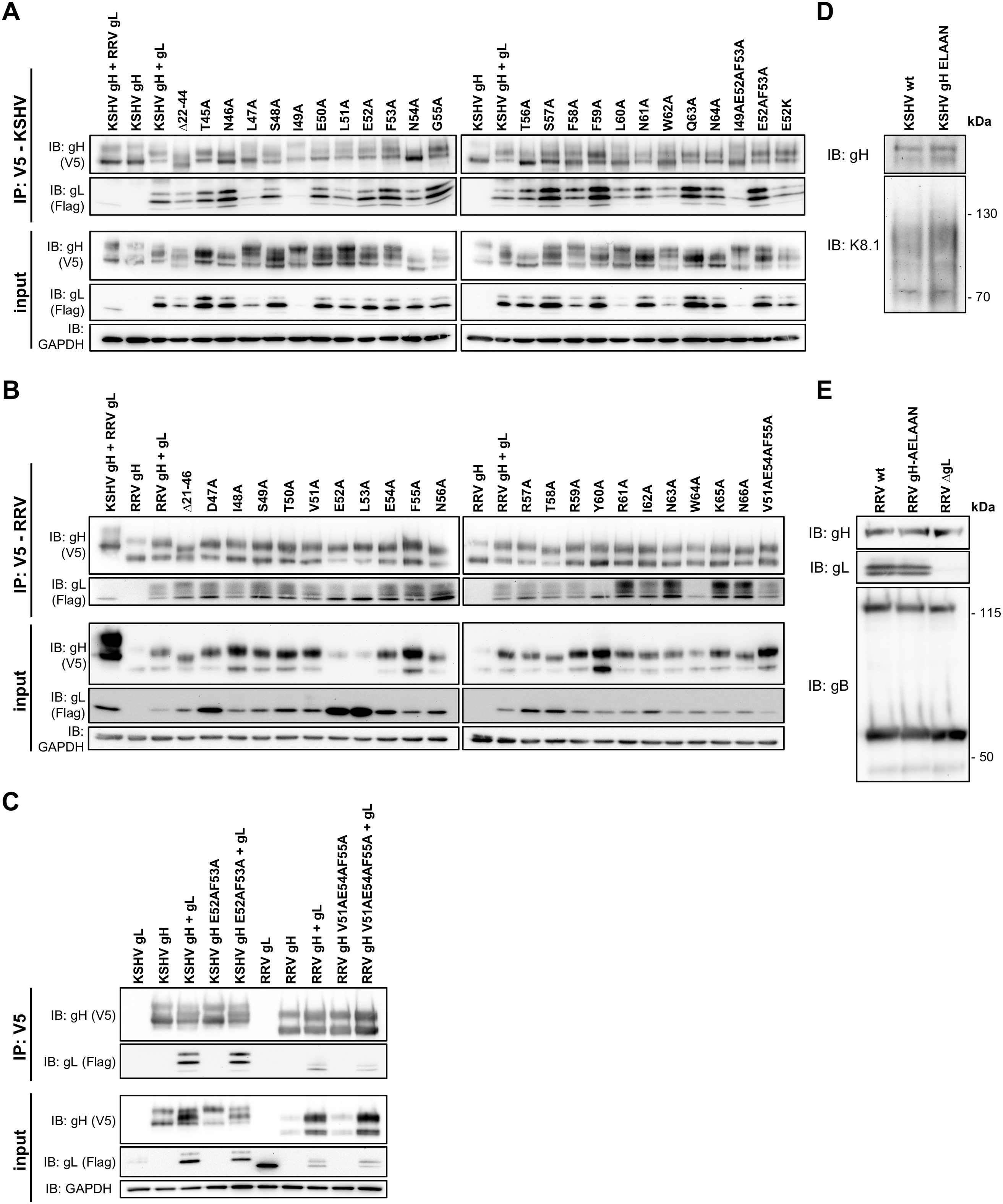

Supplement: S1 Fig — A Effects of point mutations on the stability of KSHV gH/gL complexes in the absence of recombinant Eph receptors. V5-tagged KSHV gH mutants were co-expressed with Flag-tagged KSHV gL. gH-V5/gL-Flag complexes were immunoprecipitated using monoclonal antibody to the V5-tag and precipitates were analyzed by Western blot. KSHV gH/RRV gL and KSHV gH alone serve as negative control. B Effects of point mutations on the stability of RRV gH/gL complexes in the absence of recombinant Eph receptors. V5-tagged RRV gH mutants were co-expressed with Flag-tagged RRV gL. gH-V5/gL-Flag complexes were immunoprecipitated and analyzed as in A. KSHV gH/RRV gL and RRV gH alone serve as negative control. C Point mutations in the E-L-E-F-N motif of KSHV and RRV gH do not influence stability of gH alone or of the gH/gL complex. V5-tagged KSHV gH wt, gH E52AF53A (gH-ELAAN), RRV gH wt and gH V51AE54AF55A (gH-AELAAN) were either expressed alone or co-expressed with Flag-tagged KSHV/RRV gL. gH-V5 and gH-V5/gL-Flag complexes were immunoprecipitated and analyzed as in A. D Double mutation E52AF53A in KSHV gH does not influence the incorporation of gH into the virus particle. KSHV wt, and gH-ELAAN virus preparations were analyzed by Western Blot. K8.1 was used as loading control. K8.1 runs in a diffuse molecular weight pattern due to its complex O-glycosylation. E Triple mutation V51AE54AF55A in RRV gH (gH-AELAAN) does not influence the incorporation of gH and gL into the virus particle. RRV wt, gH-AELAAN and RRV ΔgL virus preparations were analyzed by Western Blot. gB was used as loading control. Abbreviations: IP: immunoprecipitation, IB: immunoblotting. (TIF) [file ppat.1006912.s001.tif]

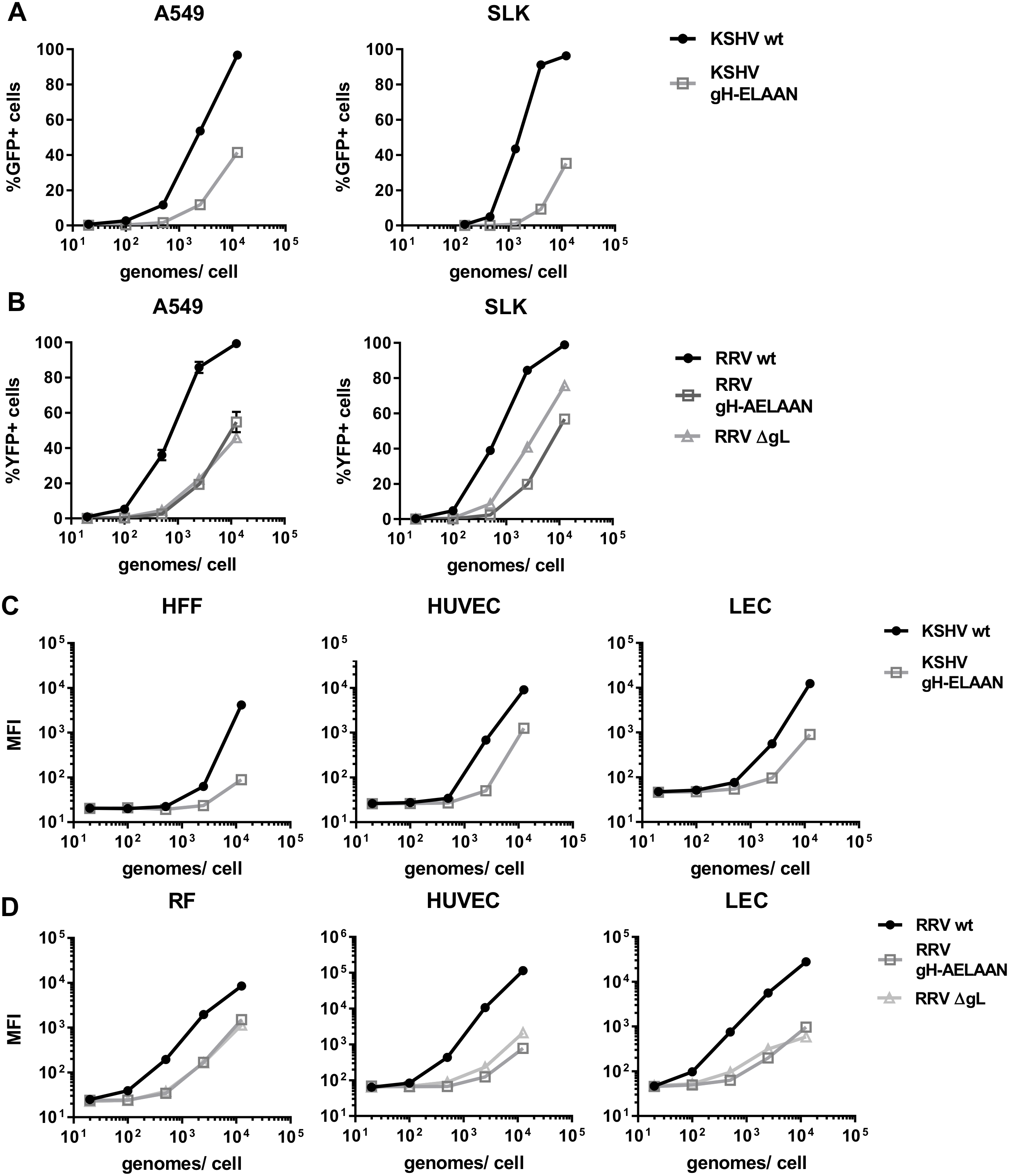

Supplement: S2 Fig — A-B Eph-binding-negative RRV and KSHV mutants exhibit a reduced specific infection on epithelial cells. Target cells were infected with KSHV wt and gH-ELAAN (A) or RRV wt, gH-AELAAN and ΔgL (B) at the indicated virus concentrations. GFP (KSHV) or YFP (RRV) expression as indicator of infection was measured by flow cytometry (triplicates, error bars indicate sd). C-D Eph-binding-negative RRV and KSHV mutants exhibit a reduced specific infection assayed by mean fluorescence intensity (MFI) of the respective reporter gene. Target cells were infected with KSHV wt and gH-ELAAN (C) or RRV wt, gH-AELAAN and ΔgL (D) at the indicated virus concentrations. GFP (KSHV) or YFP (RRV) MFI as indicator of infection was measured by flow cytometry (triplicates, error bars indicate sd). (TIF) [file ppat.1006912.s002.tif]

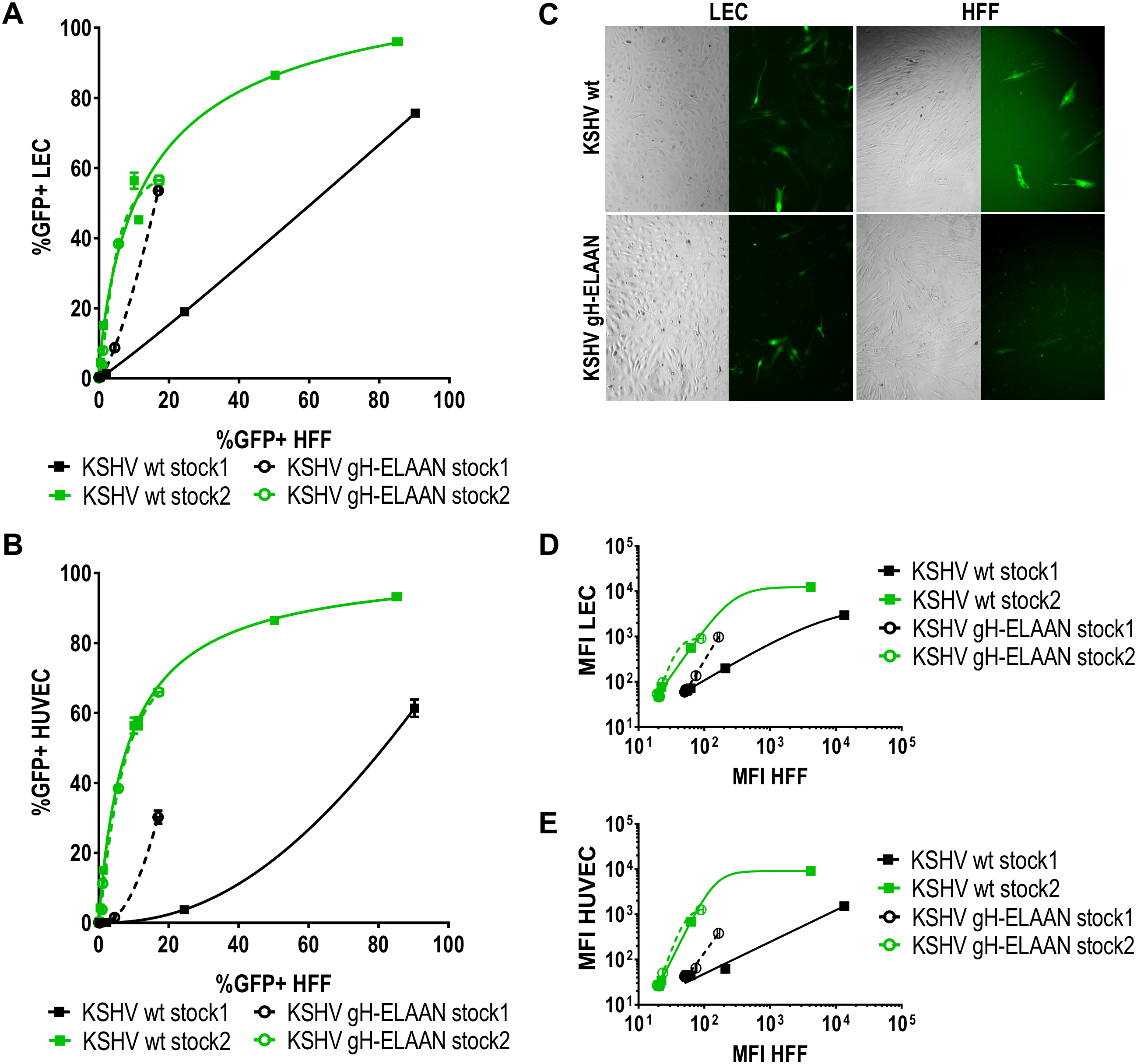

Supplement: S3 Fig — A-B Comparison of KSHV wt with KSHV gH-ELAAN infection based on GFP reporter gene-positive cells on LEC (A) or HUVEC (B) and HFF. HFF and LEC or HUVEC were infected with the same inocula of the respective virus stock, and the percentage of reporter gene-positive cells as determined by flow cytometry for each dilution was plotted. C Micrograph of HFF and LEC infected with the same inocula of wt and Eph-binding-negative KSHV. D-E Comparison of KSHV wt and KSHV gH-ELAAN infection based on MFI on LEC (D) or HUVEC (E) and HFF performed as in (A-B). (TIF) [file ppat.1006912.s003.tif]
